# Apigenin attenuates obesity-associated hepatic dysfunction and fibrosis in rats: an integrated biochemical, histological, and ex vivo dielectric study

**DOI:** 10.1186/s40360-026-01097-0

**Published:** 2026-02-20

**Authors:** Sherif Abdelmottaleb Moussa, Samir Aziz, Rehab F. Abdel-Rahman, Marawan A. Elbaset, Hany M. Fayed, Marwa E. Shabana, Fatma A. Ibrahim, Samir A. E. Bashandy

**Affiliations:** 1 National Research Centre, Department of Biochemistry, Biotechnology Research Institue, 33 El-Bohouth St., Dokki, Cairo, P.O.12622 Egypt; 2 National Research Centre, Pharmacology Department, Medical Studies Research and Clinical Institute, 33 El-Bohouth St., Dokki, Cairo, P.O.12622 Egypt; 3 National Research Centre, Department of Pathology, Medical Studies Research and Clinical Institute, 33 El-Bohouth St., Dokki, Cairo, P.O.12622 Egypt

**Keywords:** Apigenin, Obesity, Dielectric spectroscopy, Oxidative stress, NAFLD, Liver fibrosis

## Abstract

**Background:**

Apigenin (APG), a naturally occurring flavonoid, has been widely reported to exert anti-inflammatory, antioxidant, and metabolic regulatory effects. However, its therapeutic impact on obesity-associated nonalcoholic fatty liver disease (NAFLD), particularly in relation to hepatic fibrosis and tissue biophysical properties, remains incompletely characterized. This study investigated the effects of APG on biochemical, histopathological, molecular, and dielectric characteristics of the liver in a diet-induced obese rat model.

**Methods:**

Twenty-four male rats were equally divided into 4 groups (*n* = 6/each).The first (control) and second (obese) groups were not treated while the obese rats in the third and fourth groups were given 25 and 50 mg/kg APG orally respectively for 6 weeks. Inflammatory markers, oxidative stress parameters and routine liver function tests were estimated. Moreover, histological study was performed. Broadband dielectric spectroscopy was applied ex vivo to characterize frequency-dependent dielectric properties of liver tissue following uniform fixation and dehydration protocols.

**Results:**

APG treatment was associated with a dose-dependent attenuation of hepatic signal transducer and activation of transcription 3 (STAT3) and its phosphorylated (p-STAT3) levels and a significant enhancement of nuclear factor erythroid 2–related factor 2 (NRF2) expression. These molecular changes were accompanied by reductions in plasma inflammatory markers, liver function enzyme levels, and body mass index (BMI) in obese rats. APG also significantly improved hepatic redox status, as indicated by increased glutathione (GSH) and superoxide dismutase (SOD) levels and reduced malondialdehyde (MDA), reflecting decreased lipid peroxidation. Histological analyses demonstrated marked reductions in hepatic steatosis and fibrosis in APG-treated groups. Dielectric spectroscopy revealed obesity-associated alterations in tissue dielectric behavior, including reduced dielectric constant and loss, increased real impedance, and altered conductivity across frequency ranges. APG administration partially to markedly restore these dielectric parameters toward control values in a dose-dependent manner, with the higher dose exhibiting the greatest degree of normalization in impedance spectra and Nyquist plot profiles.

**Conclusions:**

Collectively, these findings indicate that apigenin ameliorates obesity-induced hepatic dysfunction through coordinated improvements in inflammatory signaling, oxidative balance, and tissue architecture. Changes in dielectric properties were interpreted as associative biophysical signatures of structural and compositional tissue remodeling, rather than direct measures of in vivo membrane electrophysiology. Within these limits, dielectric spectroscopy emerges as a complementary, non-invasive biophysical approach for monitoring hepatic tissue alterations and therapeutic response in experimental NAFLD models.

## Introduction

Obesity, arising from a chronic imbalance between energy intake and expenditure, constitutes a major global public health challenge. It is accompanied by profound metabolic disturbances, including systemic low-grade inflammation, dyslipidemia, and insulin resistance, largely driven by dysfunctional adipose tissue expansion and sustained secretion of pro-inflammatory adipokines and cytokines [1]. These metabolic abnormalities substantially increase the risk of multiple chronic diseases, among which non-alcoholic fatty liver disease (NAFLD) has emerged as one of the most prevalent and clinically significant hepatic manifestations [2]. Over the past two decades, NAFLD has increased markedly as a proportion of chronic liver diseases, with reported prevalence rising from approximately 47% to 75%, reflecting its expanding global burden in parallel with escalating obesity rates [3].

NAFLD represents a continuum of hepatic disorders, beginning with simple steatosis characterized by lipid accumulation in hepatocytes, hepatocellular injury, inflammation, and fibrotic remodeling. Approximately 20% of NAFLD patients is liable to the risk of advanced complications, including cirrhosis and hepatocellular carcinoma [4, 5]. Persistent hepatic inflammation plays a central role in this transition by promoting activation of hepatic stellate cells (HSCs), the principal fibrogenic cell population within the liver [6]. Upon activation, HSCs transdifferentiate into myofibroblast-like cells that secrete excessive extracellular matrix (ECM) components, leading to architectural distortion and fibrosis [7]. Importantly, fibrosis stage has consistently been identified as the strongest histological predictor of liver-related morbidity and mortality in patients with non-alcoholic steatohepatitis (NASH) [8–10]. Despite this clinical significance, effective anti-fibrotic therapies for NAFLD remain limited, underscoring the urgent need for novel preventive and therapeutic strategies.

In this context, increasing attention has focused on naturally occurring bioactive compounds with anti-inflammatory, antioxidant, and metabolic regulatory properties. Apigenin (5,7-dihydroxy-2-(4-hydroxyphenyl)-4 H-1-benzopyran-4-one), a dietary flavonoid abundantly present in chamomile, parsley, celery, and citrus fruits, has shown considerable therapeutic promise in preclinical models [11]. Apigenin has been reported to exert anti-inflammatory and lipid-lowering effects and to improve obesity-related metabolic disturbances. Mechanistic studies suggest that apigenin modulates hepatic lipid metabolism by downregulating lipogenic regulators such as sterol regulatory element-binding protein-1c (SREBP-1c) and acetyl-CoA carboxylase (ACC), while enhancing fatty acid oxidation through activation of peroxisome proliferator-activated receptor alpha (PPARα) and carnitine palmitoyltransferase-1 (CPT1) [11]. In parallel, apigenin attenuates oxidative stress by activating nuclear factor erythroid 2–related factor 2 (NRF2), leading to increased expression of antioxidant enzymes including superoxide dismutase (SOD), catalase, and glutathione peroxidase (GPx), while suppressing lipid peroxidation pathways such as 5-lipoxygenase (5-LOX) activity [12]. Additional evidence indicates that apigenin supports mitochondrial integrity and cellular energy homeostasis and exhibits anti-obesity and anti-diabetic effects in high-fat diet (HFD) models, improving hepatic lipid handling, glucose tolerance, and systemic metabolic control [13, 14].

Beyond biochemical and molecular assessments, there is growing interest in biophysical approaches capable of characterizing tissue-level alterations associated with metabolic liver disease. Dielectric spectroscopy (DS) is a frequency-dependent technique that measures the electrical response of biological tissues and provides information related to tissue composition, hydration, and structural organization. Increasing evidence indicates that DS can sensitively detect ex vivo alterations associated with lipid accumulation, extracellular matrix expansion, and fibrosis-related architectural remodeling in hepatic tissue [15]. Changes in dielectric parameters, including increased impedance, altered conductivity, and reduced dielectric constant, have been associated with pathological modifications in tissue composition and water distribution, often linked to oxidative stress and fibrotic ECM deposition [16]. In particular, alterations within the β-dispersion frequency range (approximately 1 kHz–10 MHz) have been reported to reflect membrane-associated polarization phenomena and fibrosis-related structural changes [17]. Importantly, in experimental settings, dielectric spectroscopy is increasingly recognized as a comparative biophysical characterization tool, rather than a direct measure of in vivo electrophysiological function. When applied under uniform tissue processing conditions, DS enables relative assessment of disease-associated structural and compositional differences and their modulation following therapeutic intervention [15–17]. Within this framework, reported changes in dielectric behavior following apigenin treatment have been interpreted as associative signatures of tissue remodeling and improved histo-biochemical status, rather than direct evidence of restored membrane ion channel activity or electrophysiological function [18].

Despite accumulating evidence supporting the metabolic and antioxidant benefits of apigenin, its influence on obesity-associated liver fibrosis, particularly when evaluated through integrated biochemical, histopathological, and biophysical approaches, remains insufficiently explored. Therefore, the present study aimed to investigate the therapeutic effects of apigenin on obesity-induced hepatic dysfunction and fibrosis in a rat model. By combining molecular and biochemical analyses with histological assessment and ex vivo dielectric spectroscopy, this work seeks to provide a comprehensive characterization of apigenin-associated hepatic remodeling and to evaluate the utility of dielectric parameters as complementary indicators of structural tissue changes during disease progression and therapeutic response.

## Materials and methods

### Experimental animals

Male Wistar rats (138–155 g, 10 weeks old) were procured and maintained at the Animal House of the National Research Centre, Egypt. Animals were housed under controlled conditions with a 12-hour light/dark cycle, ambient temperature of 25 °C, and ad libitum access to standard food and water. All procedures were conducted in accordance with the ethical guidelines approved by the National Research Centre’s Animal Care and Use Committee (Reg. No. 13060187). Experimental protocols adhered strictly to the Animal Welfare Compliance Guide for the Care and Use of Laboratory Animals (8th edition, 2011) and complied with ARRIVE guidelines.

### Chemicals

All chemicals utilized were of high analytical grade. Apigenin was obtained from Swanson, North Dakota, USA.

### Experimental design

Obesity was induced following the protocol described by [19]. Six rats were designated as the normal control group, while eighteen rats were subjected to a high-fat diet (HFD) and 25% sucrose in tap water for 16 weeks to induce obesity. Subsequently, these obese rats received either distilled water or apigenin treatment for an additional 6 weeks while having a standard diet. The HFD consisted of 42.3% carbohydrates, 17% protein, 22.5% fat, 3.2% fiber, 5% minerals, and 10% moisture. Control rats were maintained on a standard pellet diet.

Animals were randomly assigned to four groups (*n* = 6 per group):

- Group 1: Normal control rats.
- Group 2: Obese rats treated with distilled water (vehicle).
- Group 3: Obese rats orally received a treatment of 25 mg/kg apigenin [20], suspended in distilled water, administered orally for a duration of 6 weeks, and were designated as the O + APG (25 mg/kg) group.
- Group 4: Obese rats orally received a treatment of 50 mg/kg apigenin [20], suspended in distilled water, administered orally for 6 weeks, and were designated as the O + APG (50 mg/kg) group.

### Anthropometric measurements

Body mass index (BMI) and waist circumference were measured at baseline and at two-week intervals until week 6. BMI was calculated using the formula: BMI = Body weight (g) / [Body length (cm)]².

### Blood collection and plasma preparation

After a 10-hour fasting period at the end of the treatment, blood samples were collected from the retro-orbital venous plexus under light ketamine anesthesia. Plasma was separated by centrifugation and stored at -20 °C for biochemical assays.

### Liver tissue collection and preparation

Post-blood collection, rats were euthanized via cervical dislocation under ketamine anesthesia (25 mg/kg). Liver tissues were excised, weighed, and homogenized in ice-cold saline (0.9% NaCl). Homogenates were centrifuged at 3000 rpm for 10 min at 5 °C using a cooling centrifuge (Laborzentrifugen, Sigma, Germany), and supernatants were reserved for biochemical assays. Remaining tissue samples were fixed in 10% neutral-buffered formalin for histological analysis.

### Liver function assessment

Colorimetric kits from Activos-GPL (Barcelona, Spain) were used to evaluate plasma levels of albumin (Cat. No. SU001/QC014), AST (Cat. No. EZ012LQ/LIQ-155), and ALT (Cat. No. EZ016LQ/LIQ-173-M).

### Inflammatory markers

Hepatic levels of STAT-3 (Cat# SL1672Ra) and p-STAT-3 (Cat# SLD1757Ra) were measured via ELISA using kits from Sunlong Biotech Co., Ltd. (China). Plasma concentrations of MCP-1, IL-6, CRP, and TNF-α were also determined using rat-specific ELISA kits (Sunlong Biotech Co., Hangzhou, China).

### NRF2 quantification

Hepatic NRF2 levels were determined using ELISA kits (Cat# SL0985Ra, Sunlong Biotech Co., Ltd., China), following the manufacturer’s instructions.

### Oxidative stress markers

Colorimetric kits from Elabscience (Texas, USA) were employed to quantify Malondialdehyde (MDA) as a lipid peroxidation marker (Cat. No. E-BC-K025-S), Superoxide dismutase (SOD) activity (Cat. No. E-BC-K022-S), and Reduced glutathione (GSH) content (Cat. No. E-BC-K030-S).

### Histopathological evaluation

Liver samples were fixed in 10% formalin, processed, and embedded in paraffin. Sections (4 μm thick) were stained with hematoxylin and eosin (H&E) for microscopic examination. Images were captured using an Olympus CX-41 microscope with a DP-12 digital camera (Olympus Optical Co., Ltd., Tokyo, Japan).

### Fibrosis assessment

Masson’s trichrome staining (Agilent Dako, AR173, Santa Clara, CA, USA) was used for fibrosis visualization. Fibrosis was staged using the Ishak scoring system, ranging from F0 (no fibrosis) to F6 (cirrhosis) [21].

### Histomorphometric analysis

Quantitative fibrosis analysis was performed on five non-overlapping fields per specimen (×200 magnification) using the Leica Qwin DW3000 Image Analysis System (Leica Imaging Systems Ltd., Cambridge, UK). The software was programmed to measure the fibrotic area as a percentage of total tissue area, and results were reported as mean ± standard deviation.

### Biophysical analysis

#### Tissue preparation for dielectric spectroscopy

Liver samples were collected immediately after sacrifice and processed ex vivo under standardized conditions. Tissues were fixed in 10% neutral-buffered formalin for 24 h, rinsed thoroughly to remove residual fixative, and sectioned into two circular slices (1–2 mm thickness, ≥ 1.3 cm diameter) from comparable lobar regions. Samples were dehydrated sequentially in graded ethanol solutions (50–100%, 30 min each) until constant mass was achieved and then stored in sealed tubes containing 100% ethanol until analysis.

All samples were processed identically to ensure that observed dielectric differences reflected relative structural and compositional tissue changes rather than methodological variability. Dielectric measurements were therefore interpreted as comparative ex vivo biophysical indicators of tissue architecture and fibrosis-related remodeling, not as measures of in vivo membrane electrophysiology.

#### Dielectric spectroscopy

Dielectric measurements were conducted using a Broadband Dielectric Spectrometer (Novocontrol Technologies, Germany; 0.1 Hz–20 MHz) and a Keysight Impedance Analyzer (E4991B, USA; 1 MHz–3 GHz), enabling frequency-resolved analysis of impedance, conductivity, dielectric constant, and dielectric loss. Samples were measured using a custom-built cell with parallel brass electrodes (8 mm diameter) housed in a Teflon holder, with constant electrode spacing and controlled ambient temperature. The system was calibrated using standard reference materials prior to analysis. Each sample was measured in triplicate, and mean values were calculated. Dielectric parameters were derived from impedance spectra using standard relations and interpreted as comparative, frequency-dependent biophysical indicators of tissue structure and composition, including extracellular matrix remodeling, without inferring in vivo electrophysiological function.

### Statistical analysis

Data are presented as mean ± standard error of the mean (SEM). Prior to inferential analysis, data distributions were assessed for normality using the Shapiro–Wilk test, and homogeneity of variance was evaluated using the Brown–Forsythe test. For normally distributed data with equal variances, group comparisons were performed using one-way analysis of variance (ANOVA), followed by Tukey–Kramer post hoc multiple comparison tests to identify intergroup differences. All statistical analyses were conducted using appropriate statistical software, and a probability value of *p* ≤ 0.05 was considered statistically significant. Dielectric spectroscopy data were analyzed comparatively across experimental groups under identical processing conditions, with emphasis on relative intergroup differences rather than absolute parameter values.

## Results

### Effect of apigenin on body weight and BMI in obese rats

**Fig. 1:**
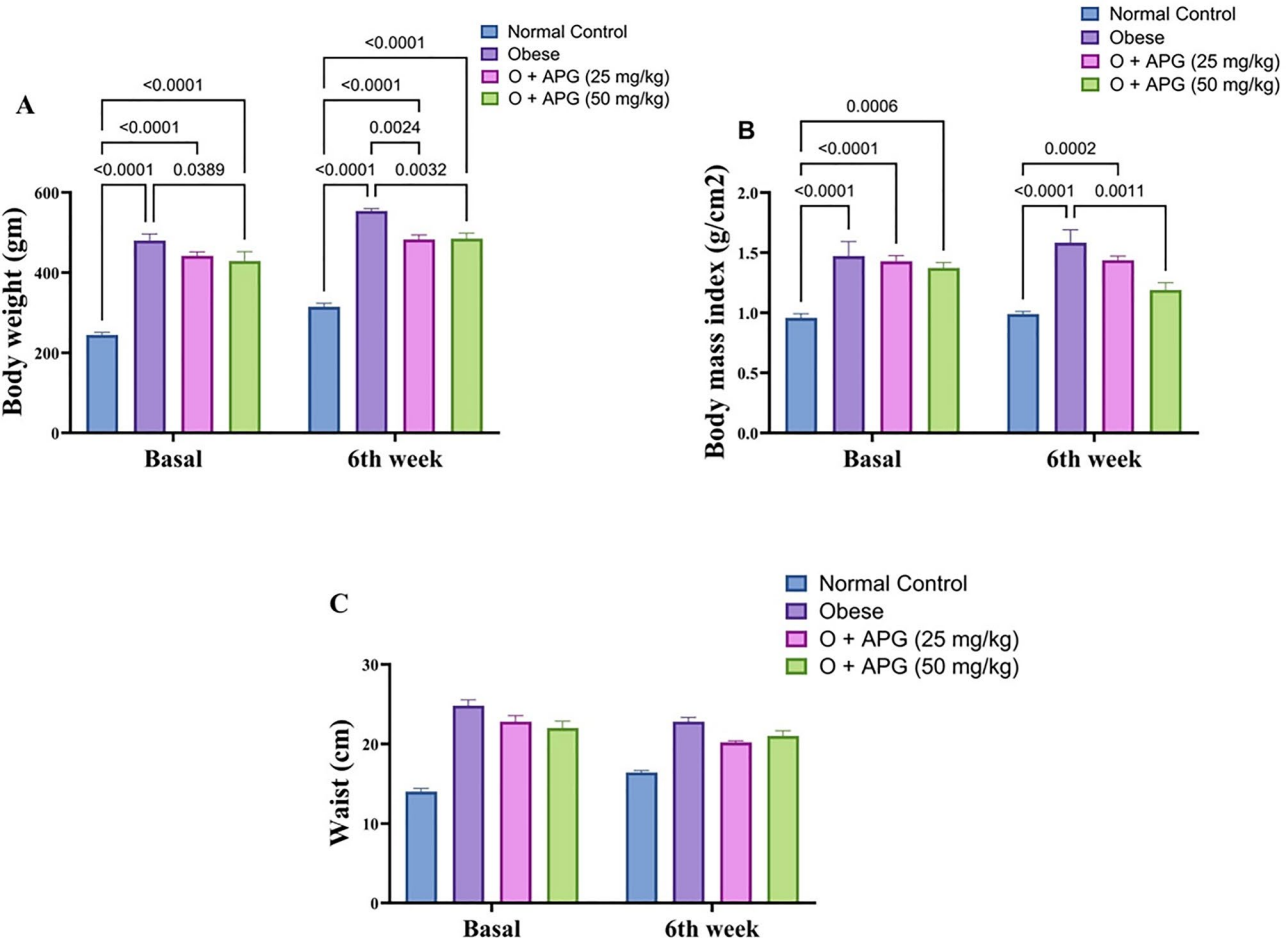
Effect of apigenin on (**A**) Body weight, (**B**) body mass index, and (**C**) waist circumference in obese rats. The bars represent the mean ± standard error of the mean (*n* = 6). Statistical analysis was conducted using one-way ANOVA, followed by Tukey–Kramer multiple comparisons within the designated time intervals, with significant levels indicated on the horizontal bars of pairwise comparisons

Obese rats exhibited a significant increase in body weight, with a 96.4% elevation relative to the normal control group. Oral administration of apigenin (APG) at doses of 25 mg/kg and 50 mg/kg resulted in reductions in body weight by 8.0% and 10.8%, respectively, compared to untreated obese rats. However, neither dose fully restored body weight to normal levels. Similarly, the body mass index (BMI) increased by 64.6% in obese rats relative to controls. Treatment with APG at 25 mg/kg and 50 mg/kg led to reductions in BMI by 8.9% and 24.7%, respectively, with the higher dose yielding a significantly greater effect than the lower dose (*p* < 0.05). These findings are illustrated in Fig. 1.Morover, waist circumference increased in all treatment groups, and no significant differences were observed between obese group and O + APG of both doses.

### Effect of apigenin on liver function parameters in obese rats

**Fig. 2:**
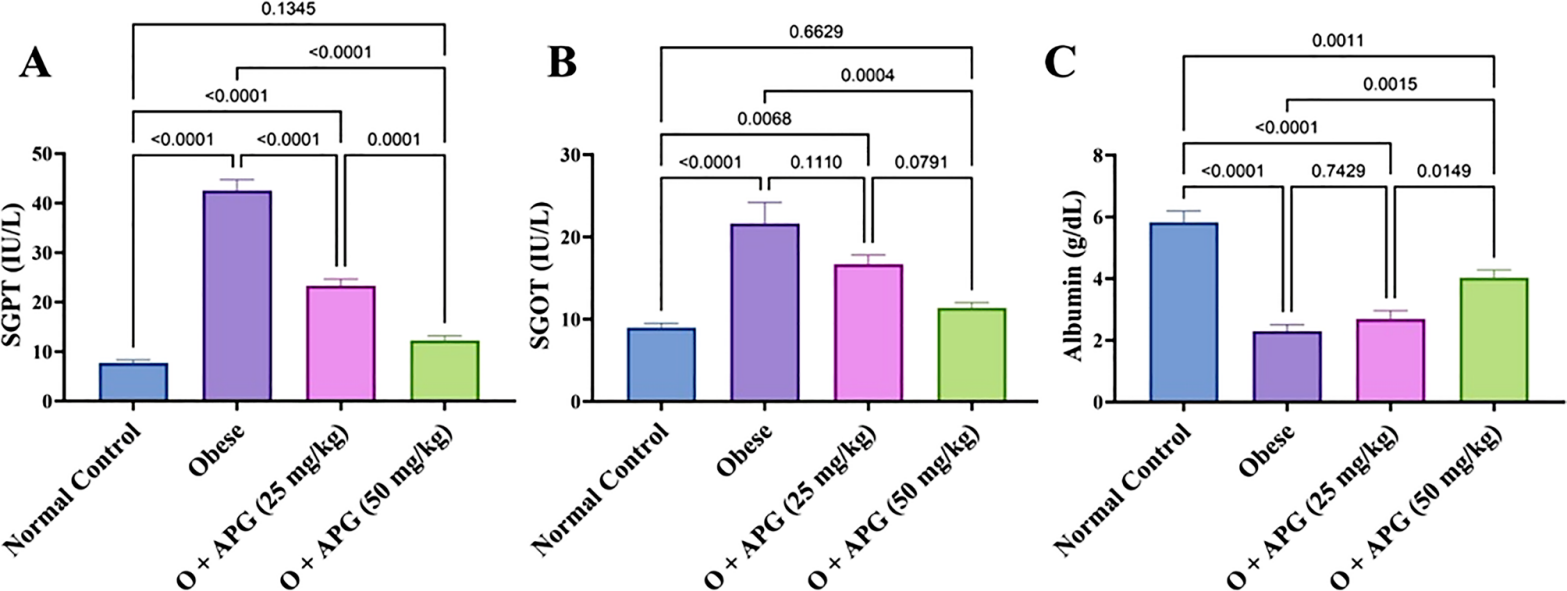
Effect of apigenin on (**A**) SGPT, (**B**) SGOT, and (**C**) albumin in obese rats. The bars represent the mean ± standard error of the mean (n = 6). Statistical analysis was conducted using one-way ANOVA, followed by Tukey–Kramer multiple comparisons within the designated time intervals, with significant levels indicated on the horizontal bars of pair­wise comparisons

Obesity markedly impaired liver function, as evidenced by substantial elevations in serum glutamic pyruvic transaminase (SGPT) and serum glutamic oxaloacetic transaminase (SGOT) levels by 452% and 140.9%, respectively accompanied by a 60.7% reduction in serum albumin levels compared to the control group. Treatment with apigenin (APG) at 25 mg/kg resulted in decreases in SGPT and SGOT levels by 45.3% and 22.9%, respectively. The higher dose of 50 mg/kg produced more pronounced effects, with reductions of 71.1% and 47.4% in SGPT and SGOT, respectively. Additionally, albumin levels increased by 17.9% with 25 mg/kg APG and by 76% with the 50 mg/kg dose. The 50 mg/kg treatment significantly outperformed the lower dose in improving all measured liver function parameters (*p* < 0.05), with albumin levels approaching those of the control group. These findings are illustrated in Fig. 2.

### Effect of apigenin on oxidative stress and inflammatory markers in obese rats

**Fig. 3:**
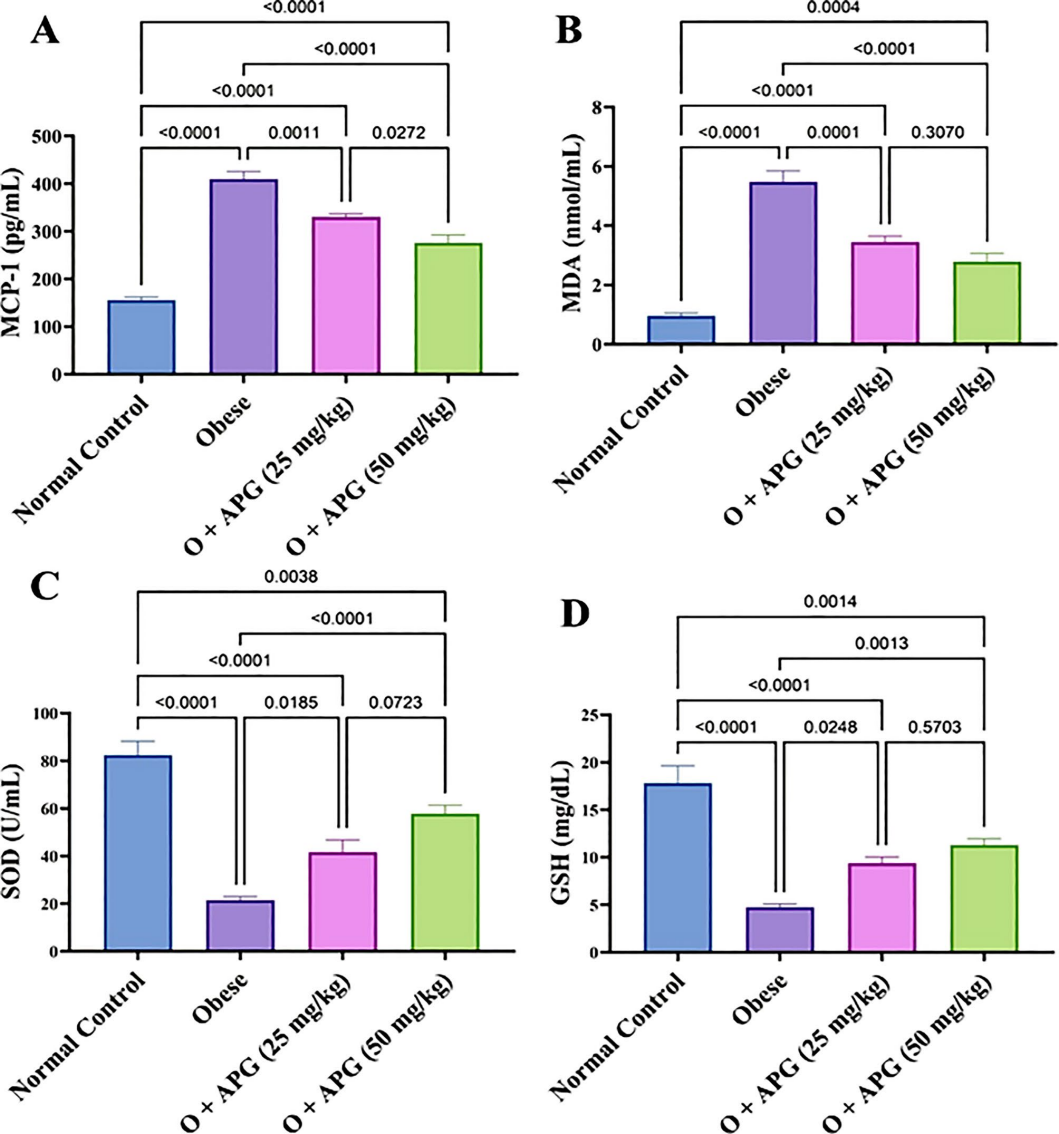
Effect of apigenin on (**A**) MCP-1, (**B**) MDA, (**C**) SOD, and (**D**) GSH in obese rats. The bars represent the mean ± standard error of the mean (*n* = 6). Statistical analysis was conducted using one-way ANOVA, followed by Tukey–Kramer multiple comparisons within the designated time intervals, with significant levels indicated on the horizontal bars of pairwise comparisons

Obesity triggered pronounced oxidative stress and systemic inflammation, as evidenced by a 163% increase in monocyte chemoattractant protein-1 (MCP-1) and a 471% elevation in malondialdehyde (MDA) levels, alongside marked reductions in antioxidant defenses—superoxide dismutase (SOD) and glutathione (GSH) levels decreased by 73.9% and 73.3%, respectively, compared to the control group. Administration of apigenin (APG) at 25 mg/kg attenuated these effects, reducing MCP-1 and MDA by 19.4% and 37%, respectively, while enhancing SOD and GSH levels by 93.8% and 97.3%. A more pronounced therapeutic effect was observed with the 50 mg/kg dose, which reduced MCP-1 and MDA by 32.7% and 49.1%, and elevated SOD and GSH levels by 169.2% and 137.7%, respectively. The higher dose of APG was significantly more effective than the lower dose (*p* < 0.05), although neither treatment fully restored these parameters to baseline levels (Fig. 3**).** Furthermore, apigenin significantly mitigated the obesity-induced elevations in pro-inflammatory cytokines, including interleukin-6 (IL-6), C-reactive protein (CRP), and tumor necrosis factor-alpha (TNF-α), as detailed in Table 1.

**Table 1:** Effect of apigenin on plasma inflammatory markers in obese rats

Group	Control	O	O + APG 25 mg/Kg	O + APG 50 mg/Kg
Parameter				
IL6(Pg/ml)	13.00 ± 0.97	53.83 ± 2.58*	26.33 ± 1.29*@	16.00 ± 1.05@#
TNF-α(ng/L)	65.00 ± 4.41	246.67 ± 15.76*	146.67 ± 10.78*@	75.00 ± 3.33@#
CRP(ng/ml)	2.53 ± 0.26	13.13 ± 0.70*	7.32 ± 0.29*@	4.18 ± 0.28@#

Each value is the mean ± SE, *n* = 6 .Statistical analysis was performed using one-way ANOVA followed by Tukey-Kramer multiple comparisons test. (^⋆^ vs. control ^@^ vs. obese group,^#^ vs. O + APG 25 mg/Kg) at *p* < 0.05.O: Obese, APG: Apigenin

### Effect of apigenin on hepatic STAT3 signaling and NRF2 expression in obese rats

**Fig. 4:**
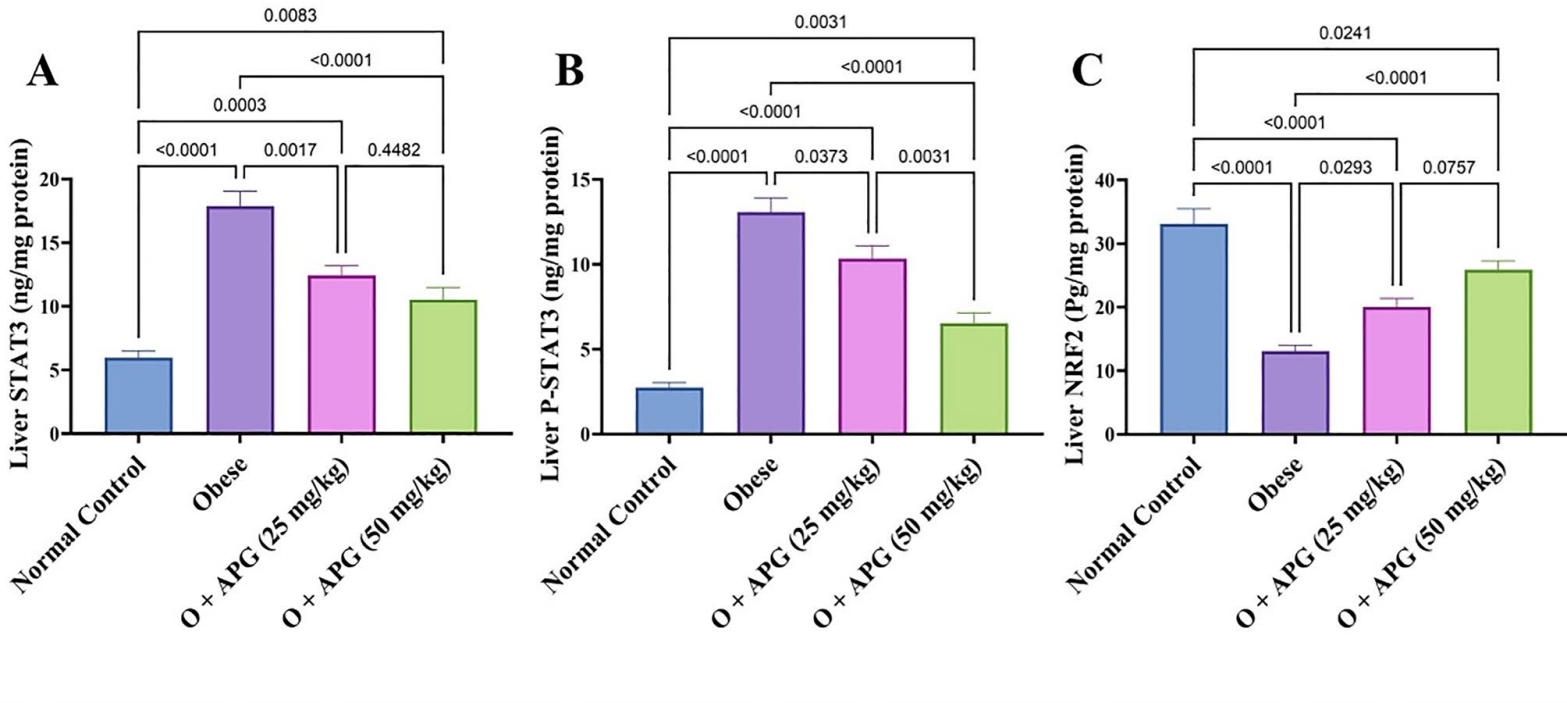
Effect of apigenin on liver content of (**A**) STAT3, (**B**) p-STAT3, and (**C**) NRF2 in obese rats. The bars represent the mean ± standard error of the mean (*n* = 6). Statistical analysis was conducted using one-way ANOVA, followed by Tukey–Kramer multiple comparisons within the designated time intervals, with significant levels indicated on the horizontal bars of pairwise comparisons

Obesity markedly activated the hepatic STAT3 signaling pathway, with total STAT3 and phosphorylated STAT3 (p-STAT3) levels increasing by 198.5% and 377%, respectively, compared to normal controls. Concurrently, nuclear factor erythroid 2–related factor 2 (NRF2), a key regulator of cellular antioxidant defense, was suppressed by 60.5%. Treatment with apigenin (APG) at a dose of 25 mg/kg resulted in moderate reductions in STAT3 and p-STAT3 levels by 30.4% and 21%, respectively, and enhanced NRF2 expression by 53.3%. The higher dose of 50 mg/kg APG elicited a more pronounced response, reducing STAT3 and p-STAT3 levels by 41% and 50%, respectively, and elevating NRF2 levels by 98.4%. The 50 mg/kg dose produced significantly greater improvements across all parameters **(***p* < 0.05), although full normalization of these markers was not achieved **(**Fig. 4).

### Histological findings

**Fig. 5:**
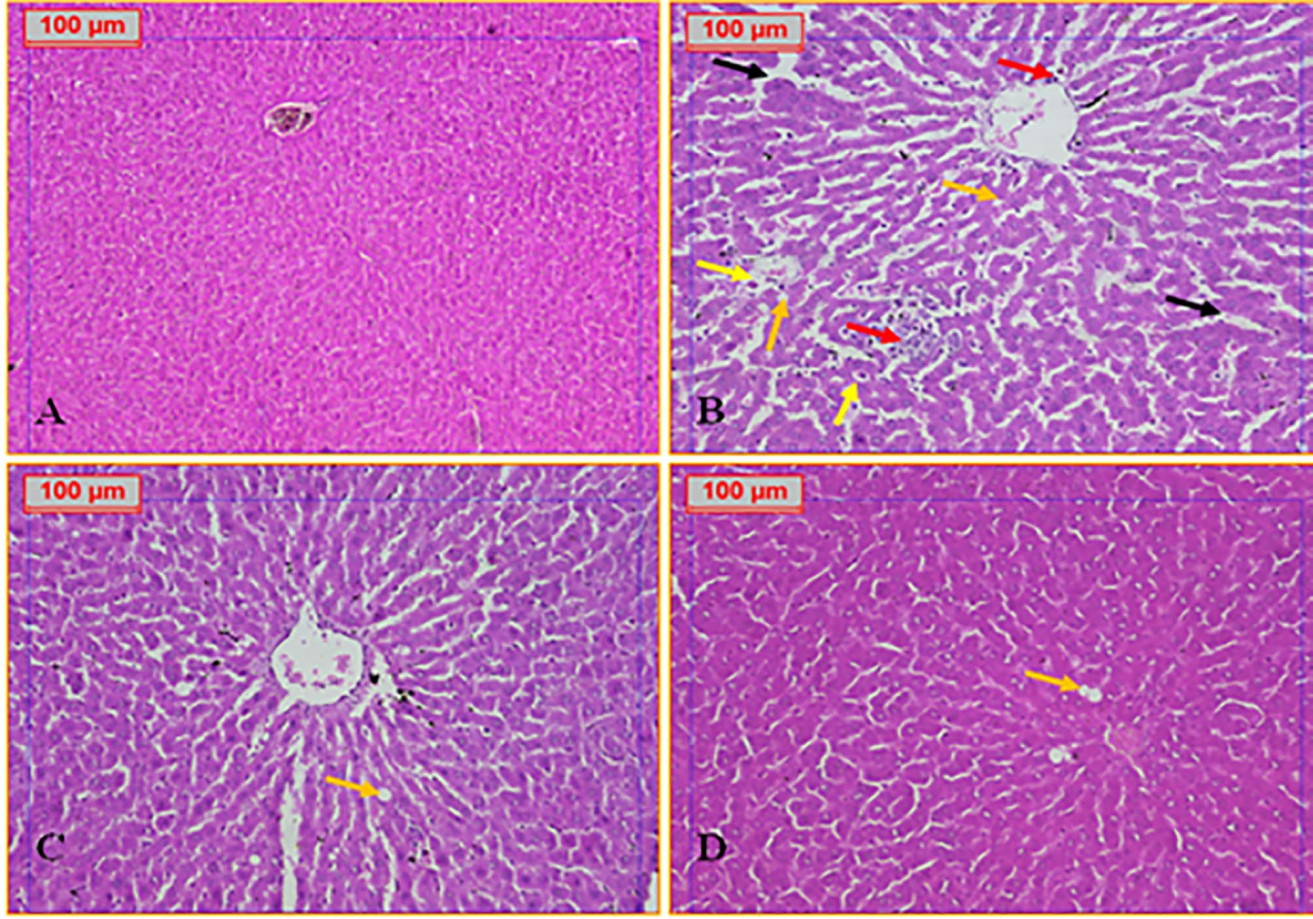
**A**: Photomicrograph of Normal Control group showing normal structure of the classical hepatic lobule. **B**: Photomicrograph of hepatic tissue of Obese group showing marked dilated congested central vein (star), hepatocytes with multiple scattered lipid droplets (steatosis) (orange arrow), scattered hepatocytes hydropic degeneration (yellow arrow), dilated sinusoidal spaces with hemorrhage (black arrow) and scattered inflammatory cells (red arrow). **C**: Photomicrograph of hepatic tissue of Obese + APG (25 mg/kg) group showing dilated central vein with minimal steatosis orange arrow), and mild dilated sinusoidal spaces **D**: Photomicrograph of hepatic tissue of Obese + APG (50 mg/kg) group showed normal hepatic tissue with minimal fat cells deposits (orange arrow) (H&E, x200)

Histological examination of liver tissues revealed normal hepatic architecture in the negative control group. The liver parenchyma was organized into classical hepatic lobules composed of hepatocyte cords radiating from a central vein toward the periphery, with intact sinusoidal architecture (Fig. 5A). In contrast, the obese positive control group exhibited pronounced histopathological alterations, including distorted lobular architecture, dilated central veins, leukocytic infiltration, scattered lipid droplets, markedly dilated sinusoidal spaces, some with hemorrhagic foci and hepatocellular degeneration, such as hydropic changes (Fig. 5B). Treatment with apigenin at a dose of 25 mg/kg resulted in notable histological improvement. Liver sections displayed mild sinusoidal dilatation, fewer lipid-laden cells, and largely preserved hepatocyte morphology (Fig. 5C). The group treated with 50 mg/kg apigenin demonstrated near-normal hepatic architecture, with minimal fat accumulation and restoration of tissue integrity (Fig. 5D).

**Fig. 6:**
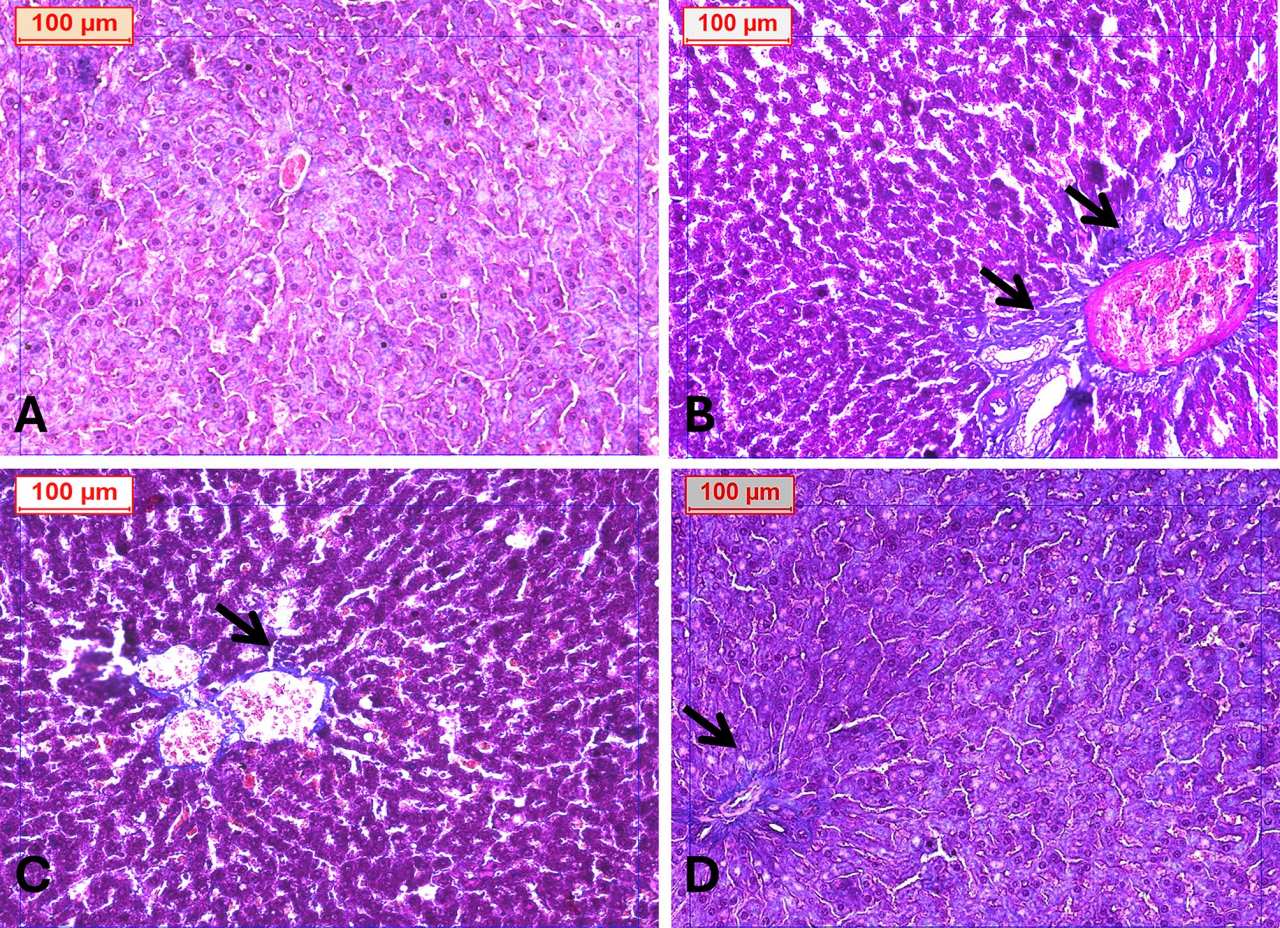
(**A**) Photomicrograph of liver tissue from the normal control group showing preserved hepatic architecture with no evidence of fibrosis. (**B**) Liver section from the obese group displaying extensive fibrotic deposition surrounding the portal tract. (**C**) Liver tissue from the Obese + APG (25 mg/kg) group exhibiting mild fibrosis localized around the central vein. (**D**) Liver section from the Obese + APG (50 mg/kg) group demonstrating nearly normal hepatic architecture with minimal fibrosis around the portal vein (arrow indicates fibrotic areas). *Masson’s Trichrome stain*,* original magnification ×200*

**Fig. 7:**
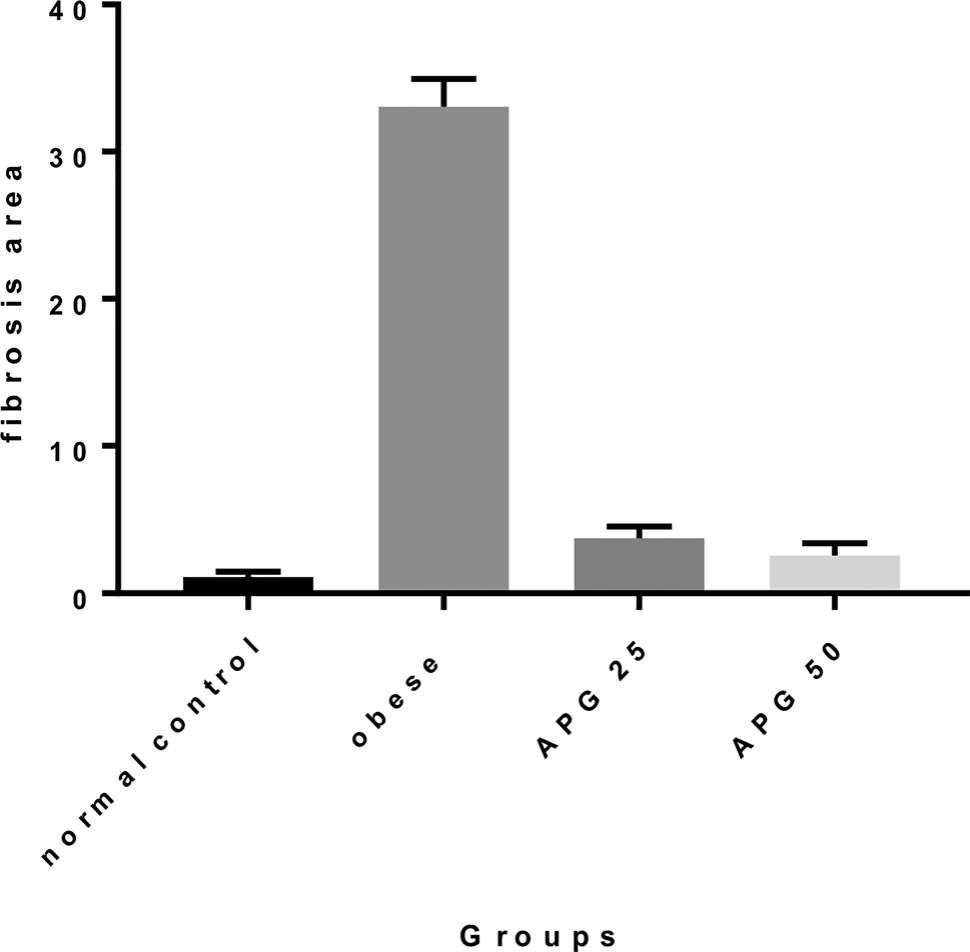
Effect of APG on obese liver. Values are expressed as mean ± SD. Statistical analysis was carried out by one way ANOVA followed by Tukey’s multiple comparisons test against control group. APG-25 (Apigenin 25 mg/kg), APG-50 (Apigenin 50 mg/kg)

Fibrosis assessment using Masson’s Trichrome staining and graded according to the Ishak scoring system further supported these findings. The negative control group exhibited no fibrosis (F0) (Fig. 6A). The obese group showed severe fibrosis, particularly pericentral and periportal (F4) (Fig. 6B). Rats treated with 25 mg/kg apigenin exhibited moderate fibrosis (F2) (Fig. 6C), while those receiving 50 mg/kg showed only minimal fibrotic changes localized around the central vein (F1) (Fig. 6D). The Fig. 7 illustrated the fibrosis scoring in different treatment groups.The treatment of obese rats with with 25 mg or 50 mg/kg apigenin reduced the fibrosis scoring significantly.

### Dielectric spectroscopy of hepatic tissues

**Fig. 8:**
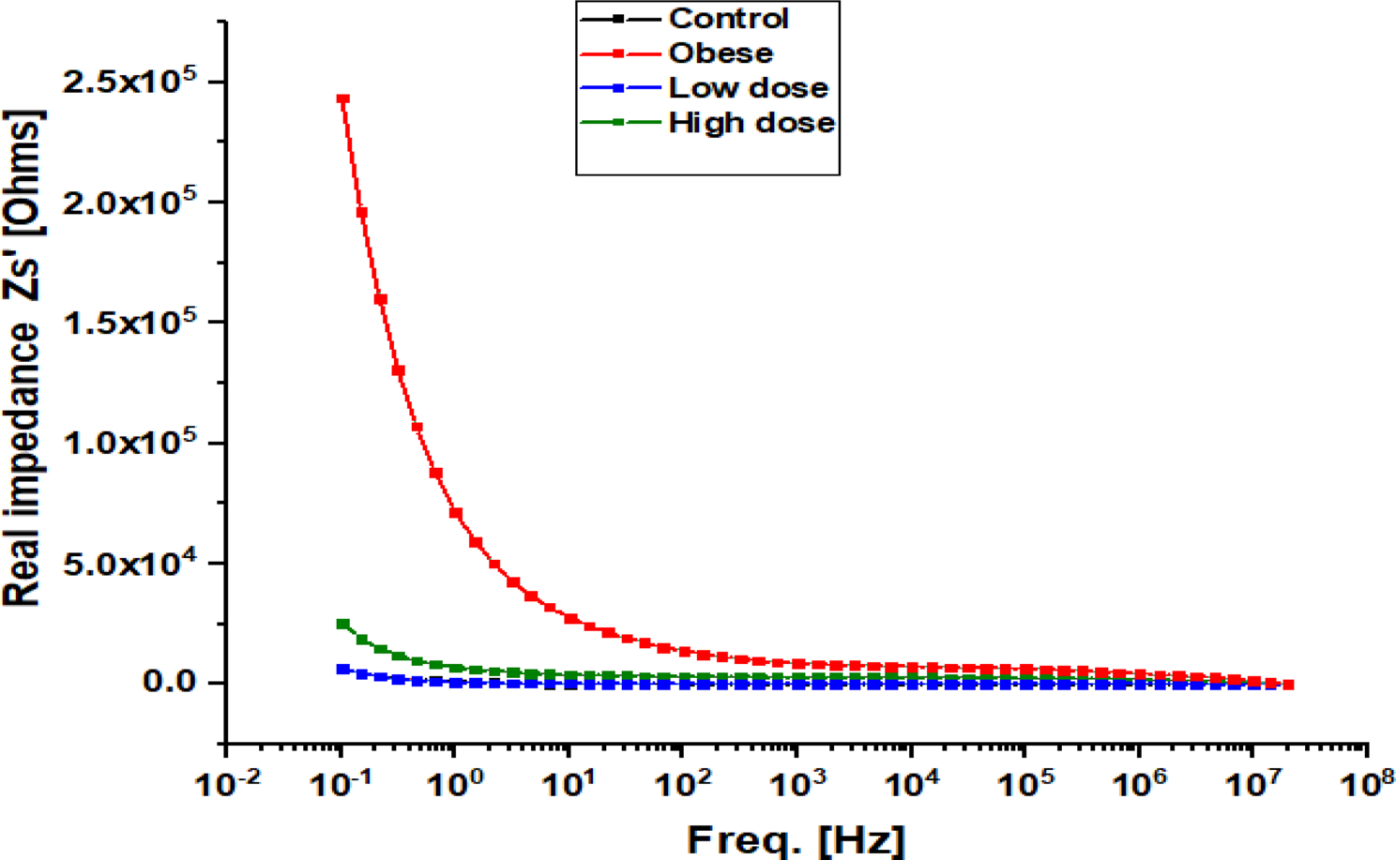
Frequency-dependent real impedance of hepatic tissue in Obese Rats Treated with Low and High Doses of Apigenin Compared to the Control Group

Figure 8 shows the frequency-dependent variation of real impedance (Zs′) across all groups. Impedance decreased with increasing frequency, consistent with the typical electrical behavior of biological tissues. The Obese group exhibited the highest impedance values, particularly at low frequencies (< 10³ Hz; Zs′ > 2.5 × 10⁵ Ω), while the Control group showed the lowest values. Apigenin treatment reduced impedance in a dose-dependent manner, with the Low-dose group showing partial reduction and the High-dose group approaching control values.

**Fig. 9:**
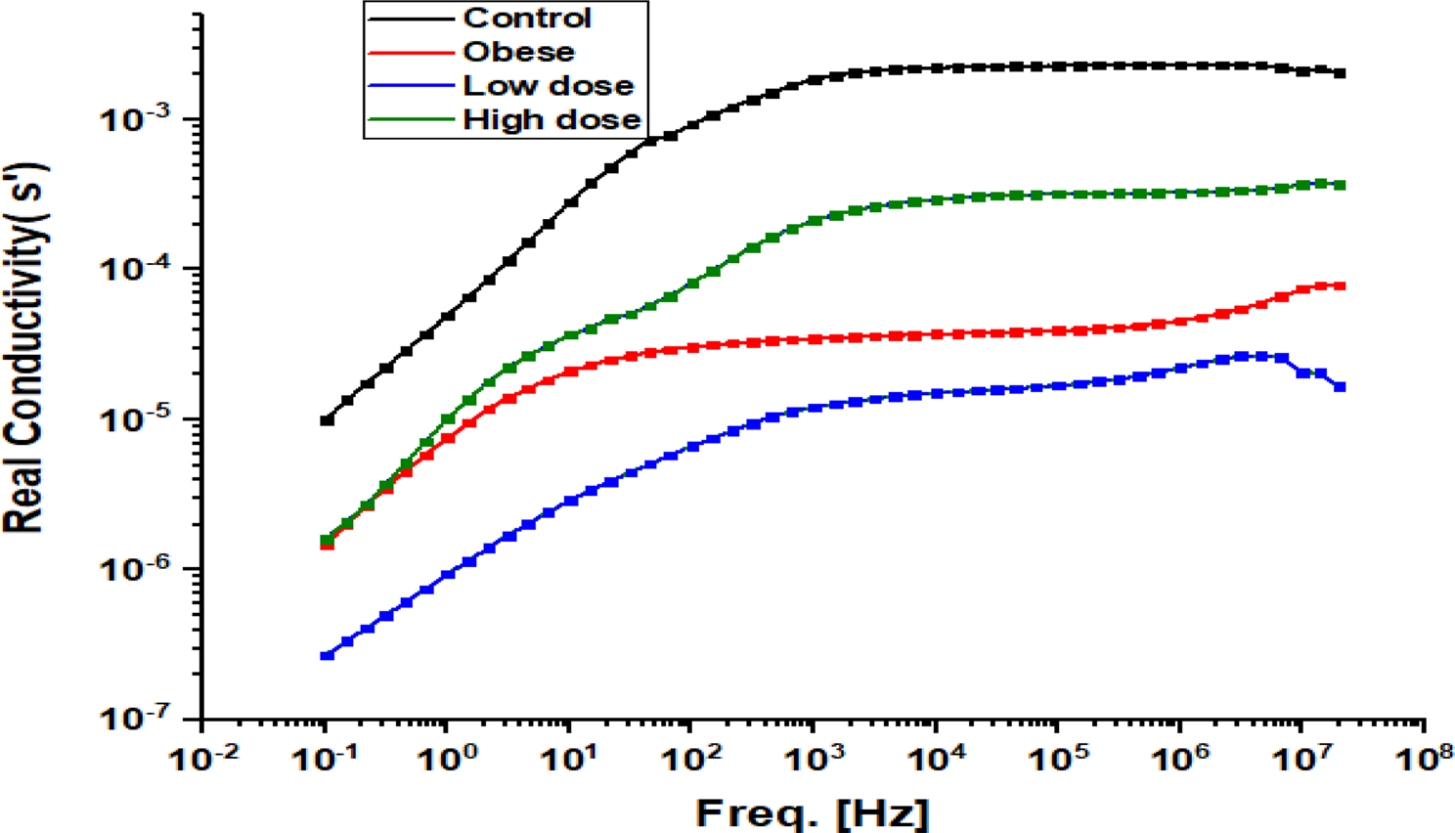
Frequency-dependent real conductivity (σ’) of hepatic tissue in Obese Rats Treated with Low and High Doses of Apigenin Compared to the Control Group

Figure 9 presents the real conductivity (σ′) of liver tissues as a function of frequency. In all groups, conductivity increased with frequency, displaying a dispersive pattern consistent with heterogeneous biological materials. At higher frequencies, σ′ values gradually approached a plateau, suggesting saturation of conductive pathways. The Control group exhibited relatively higher conductivity across the frequency range. The Obese group displayed altered conductivity behavior, particularly at lower frequencies, indicating obesity-associated changes in tissue composition and charge transport characteristics. The Low-dose APG group showed reduced conductivity values across most frequencies compared with both Control and Obese groups. In contrast, the High-dose APG group exhibited intermediate conductivity values, lying between those of the Obese and Control groups. At higher frequencies, differences among groups became less pronounced, reflecting reduced sensitivity of conductivity to structural heterogeneity in this range.

**Fig. 10:**
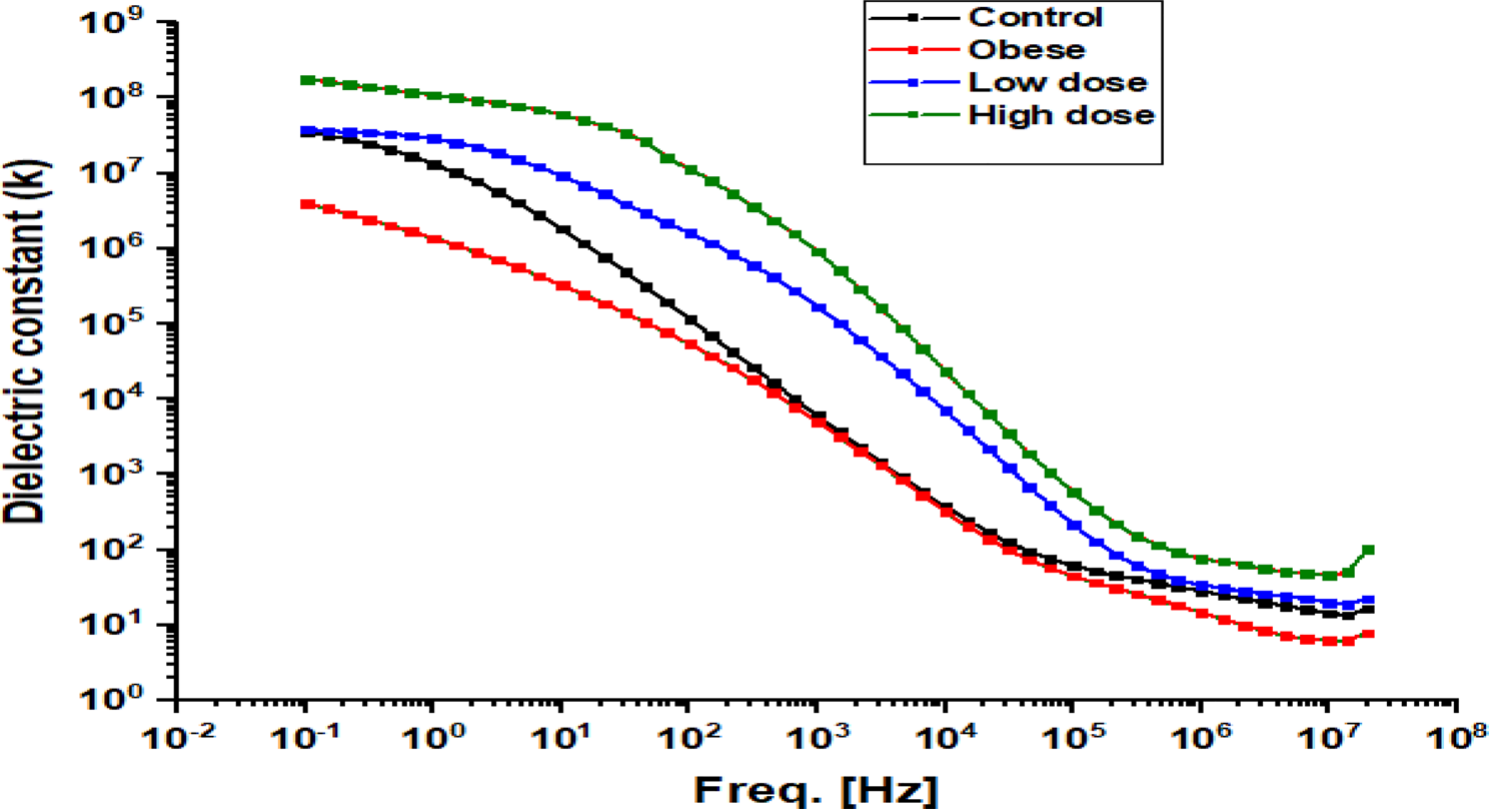
Frequency-dependent dielectric constant of hepatic tissue in Obese Rats Treated with Low and High Doses of Apigenin Compared to the Control Group

Figure 10 shows the variation in the dielectric constant (k) across frequencies. For all experimental groups, the dielectric constant was highest at low frequencies (< 10³ Hz), indicating strong polarization effects, and decreased progressively as frequency increased. The Obese group consistently exhibited lower dielectric constant values compared with the Control group across the entire frequency range, suggesting reduced polarizability associated with obesity-related tissue alterations. Both apigenin-treated groups displayed increased dielectric constant values relative to the Obese group. The Low-dose APG group showed higher dielectric constant values than both Control and Obese groups, while the High-dose APG group demonstrated the highest dielectric constant across all frequencies. These trends indicate that apigenin treatment is associated with modifications in dielectric polarization behavior, likely reflecting changes in tissue composition and structural organization.

**Fig. 11:**
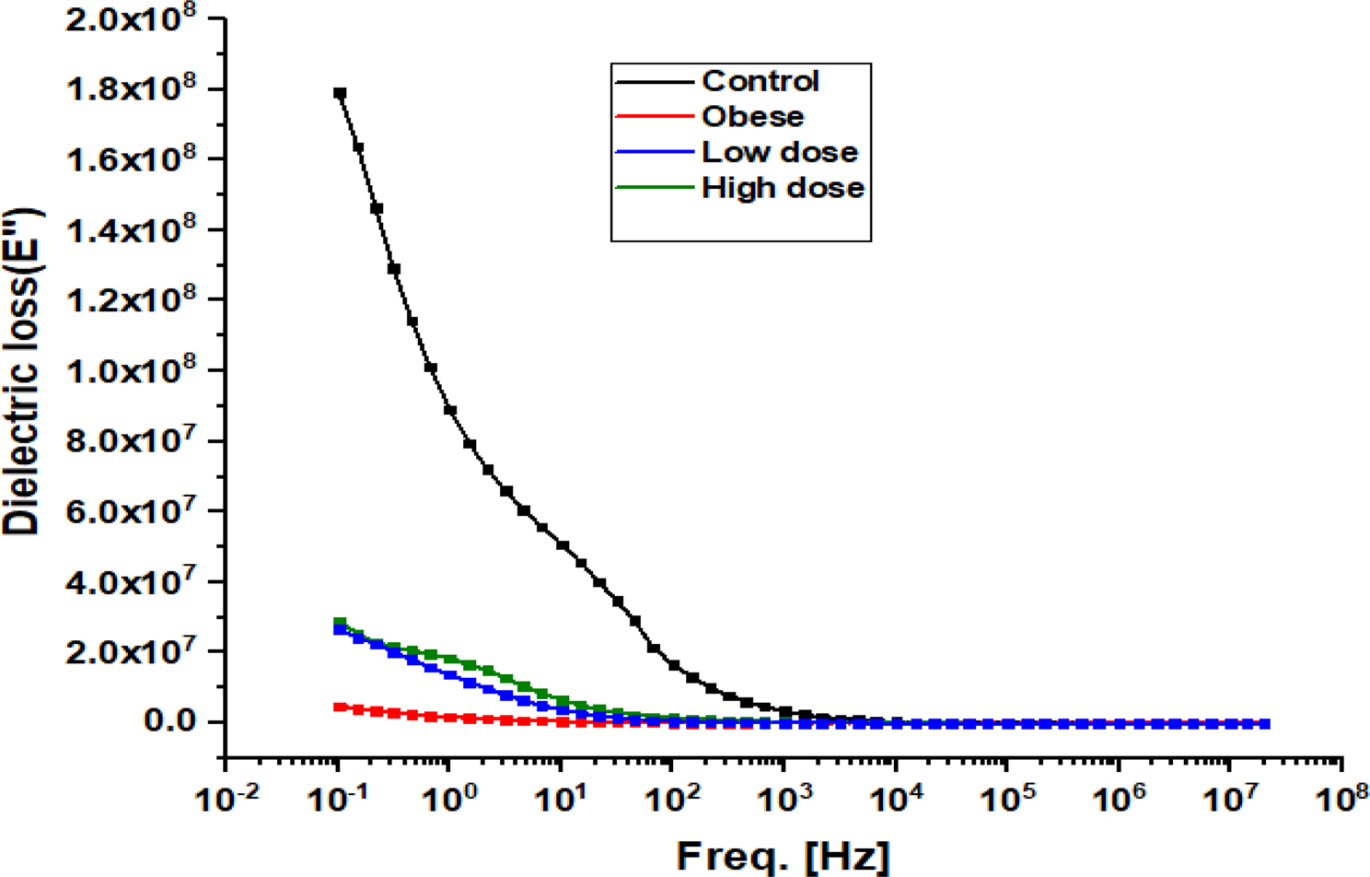
Frequency-dependent dielectric loss of hepatic tissue in Obese Rats Treated with Low and High Doses of Apigenin Compared to the Control Group

Figure 11 depicts the frequency-dependent dielectric loss (εʺ), representing energy dissipation within liver tissue arising from resistive conduction and polarization relaxation processes. Dielectric loss was highest at low frequencies (< 10³ Hz) and decreased steadily with increasing frequency in all groups. The Control group exhibited the greatest dielectric loss at low frequencies, reaching approximately 1.8 × 10⁸ at 10⁻¹ Hz. In contrast, the Obese group showed markedly lower dielectric loss values across the frequency spectrum. The Low-dose APG group demonstrated increased dielectric loss relative to the Obese group but remained below Control levels at lower frequencies. The High-dose APG group exhibited a further increase in dielectric loss, with values closely approaching those observed in the Control group, indicating partial normalization of energy dissipation behavior.

**Fig. 12:**
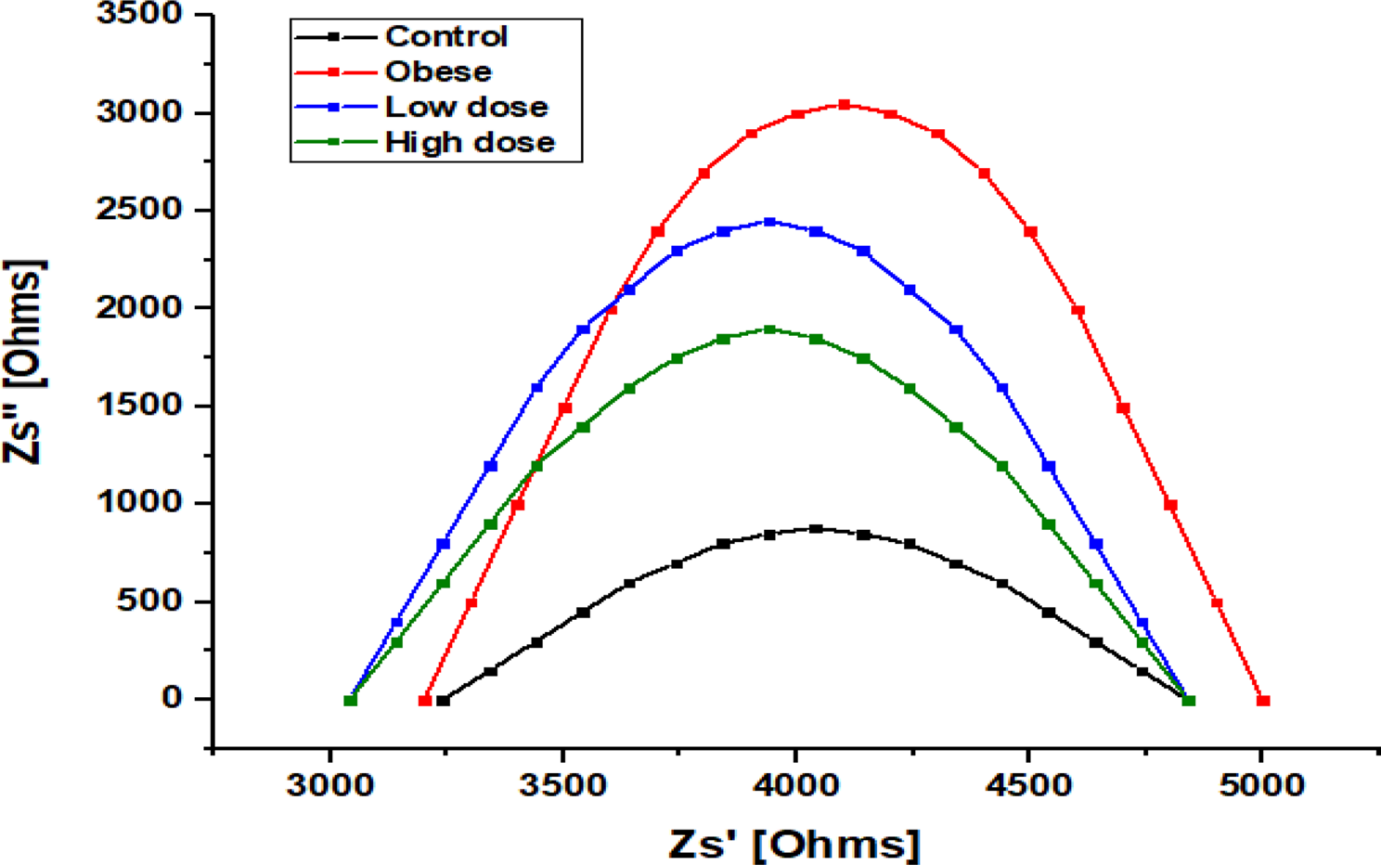
Nyquist plot of impedance spectra of hepatic tissue in Obese Rats Treated with Low and High Doses of Apigenin Compared to the Control Group

Figure 12 presents Cole–Cole (Nyquist) plots derived from impedance measurements, illustrating the dielectric relaxation behavior of liver tissues across experimental groups. All groups exhibited characteristic semicircular arcs, reflecting the dispersive and heterogeneous nature of biological tissues. The apex of each semicircle corresponds to the maximum imaginary impedance (Zsʺ), associated with capacitive and polarization-related processes. The Control group displayed the smallest semicircle, indicative of lower overall impedance. In contrast, the Obese group exhibited the largest semicircular arc, reflecting increased impedance and altered relaxation behavior. Both APG-treated groups showed intermediate profiles, with a clear dose-dependent shift toward reduced semicircle size. The High-dose APG group demonstrated a more pronounced reduction in arc magnitude than the Low-dose group, approaching the Control profile, consistent with a stronger effect of higher apigenin dosage.

## Discussion

The present study provides an integrated evaluation of the therapeutic potential of apigenin (APG) in mitigating obesity-associated hepatic dysfunction, oxidative stress, inflammation, and fibrosis. By combining biochemical, molecular, histopathological, and biophysical assessments, this work offers a comprehensive characterization of APG-associated hepatic remodeling in a diet-induced obese rat model. Across multiple experimental endpoints, APG treatment produced dose-dependent improvements in metabolic indices, antioxidant capacity, inflammatory signaling, and liver architecture. These effects were accompanied by consistent modifications in dielectric parameters, which were interpreted as associative biophysical signatures of structural and compositional tissue changes, rather than direct measures of in vivo electrophysiological function [22, 23].

Consistent with established obesity models, rats fed a high-fat/high-sucrose diet exhibited significant increases in body weight and body mass index (BMI), reflecting altered energy balance and adiposity [19, 24]. Apigenin administration resulted in a significant reduction in both parameters, suggesting a beneficial effect on systemic metabolic status. These findings are in agreement with previous studies demonstrating that APG can attenuate weight gain and improve anthropometric indices in experimental obesity, potentially through modulation of lipid metabolism and energy expenditure [25, 26]. Oxidative stress increases with increasing BMI [27].

Obesity-induced hepatic oxidative stress was a prominent feature in the current model, as evidenced by elevated malondialdehyde (MDA) levels and marked depletion of endogenous antioxidant defenses, including glutathione (GSH) and superoxide dismutase (SOD)) [28]. These alterations are well documented in NAFLD and contribute to lipid peroxidation, protein oxidation, and progressive cellular injury [29, 30]. In the present study, APG treatment significantly restored antioxidant balance in a dose-dependent manner, indicating effective attenuation of oxidative damage. These biochemical improvements were paralleled by molecular evidence of enhanced nuclear factor erythroid 2–related factor 2 (NRF2) expression, a key regulator of cellular antioxidant responses [31–34]. The ability of APG to activate NRF2 signaling has been reported previously and supports its role in restoring hepatic redox homeostasis under metabolic stress [35].

In parallel, inflammatory markers including IL-6, TNF-α, and C-reactive protein (CRP) were significantly elevated in obese rats, reflecting the chronic inflammatory milieu characteristic of nonalcoholic steatohepatitis (NASH). Apigenin administration markedly suppressed these cytokines in a dose-dependent fashion, with the higher dose (50 mg/kg) producing near-normalization. This anti-inflammatory effect was accompanied by reduced phosphorylation of STAT3, a central mediator of IL-6–driven inflammatory and fibrogenic signaling [21, 36]. Oxidative stress (OS) can stimulate the production of proinflammatory cytokines and chemokines, attracting immune cells and promoting inflammation in the liver [37].OS also promotes the activation of Hepatic Stellate Cells which can produce extracellular matrix and contribute to the development of liver fibrosis [38].

Together, these findings support the concept that APG mitigates obesity-induced hepatic inflammation through coordinated modulation of redox-sensitive and cytokine-driven pathways.

Histopathological analyses corroborated these molecular and biochemical findings, demonstrating a clear reduction in hepatic steatosis and fibrosis following APG treatment. Given that fibrosis stage is the strongest predictor of liver-related morbidity and mortality in NASH, these structural improvements underscore the therapeutic relevance of APG in obesity-related liver disease [31]. Importantly, the observed histological recovery was accompanied by systematic changes in dielectric parameters, including reductions in real impedance (Zs′), modulation of conductivity (σ′), and restoration of dielectric constant (ε′) and dielectric loss (ε″) toward control-like profiles (Figs. 8, 9, 10 and 11). Under the standardized fixation and dehydration conditions employed in this study, these dielectric changes are interpreted as comparative indicators of tissue architecture, extracellular matrix composition, and macromolecular organization, rather than direct evidence of restored membrane electrophysiology [39].

Dielectric constant and dielectric loss are commonly associated with polarization capacity and energy dissipation in heterogeneous biological tissues. In the present model, obesity was associated with reduced ε′ and ε″, suggesting diminished polarization efficiency and altered macromolecular organization, likely reflecting lipid accumulation, oxidative damage, and fibrotic ECM deposition [40]. Apigenin treatment, particularly at the higher dose, significantly increased both parameters, indicating improved dielectric polarization behavior. These changes are consistent with structural remodeling and improved tissue composition, rather than direct modulation of ion channel activity. Similar dielectric trends have been reported for other flavonoids, such as quercetin, where improvements in dielectric behavior were associated with membrane stabilization and reduced oxidative injury [41].

Nyquist (Cole–Cole) plot analysis further supported these findings by illustrating differences in relaxation behavior among experimental groups. Obese rats exhibited enlarged semicircular arcs, reflecting increased impedance and altered relaxation dynamics. In contrast, APG-treated groups showed reduced arc magnitude in a dose-dependent manner, approaching the control profile. These changes are consistent with improved tissue homogeneity and reduced resistive barriers associated with fibrosis and ECM accumulation [42, 43]. Importantly, these observations are interpreted as biophysical correlates of histological remodeling, rather than direct measures of cellular electrophysiological recovery.

The coordinated improvement in dielectric parameters closely paralleled reductions in oxidative stress and inflammatory signaling. In particular, the high-dose APG group exhibited marked decreases in MDA alongside restoration of GSH and SOD levels, accompanied by upregulation of NRF2 and downregulation of phosphorylated STAT3 [44]. These molecular corrections coincided with histological regression of fibrosis and normalization of dielectric behavior, highlighting the interdependence of redox balance, tissue structure, and biophysical properties.

Notably, dielectric improvements were also evident in the low-dose APG group, despite the presence of moderate residual fibrosis histologically [45]. This suggests that dielectric spectroscopy may be sensitive to early or partial tissue remodeling that precedes complete structural normalization. As such, dielectric measurements may provide complementary information regarding therapeutic response and tissue status when used alongside conventional biochemical and histological assessments [46].

Previous studies have demonstrated that reduced dielectric constant is associated with lipid accumulation and altered membrane organization, while increased low-frequency conductivity reflects extracellular ion redistribution and edema in damaged tissues [47–49]. The ability of APG to modulate these parameters in parallel with improvements in liver enzymes, BMI, and inflammatory markers reinforces its systemic hepatoprotective effects in obesity-associated liver disease [22, 50–52].

Finally, the frequency-dependent nature of the dielectric changes observed in this study provides insight into the complexity of hepatic recovery. Alterations at lower frequencies likely reflect changes in extracellular matrix composition and interfacial polarization, whereas higher-frequency responses may be influenced by intracellular structural organization. Together, these findings underscore the multifactorial actions of apigenin in attenuating oxidative, inflammatory, and fibrotic processes in NAFLD, while positioning dielectric spectroscopy as a complementary, ex vivo biophysical approach for assessing tissue remodeling and therapeutic efficacy under controlled experimental conditions [28, 53–55].

## Conclusions

This study shows that apigenin is associated with significant improvements in obesity-induced hepatic oxidative stress, inflammation, and fibrotic remodeling in rats. It seems that the decrease of body weight and BMI in obese rats by apigenin can participate in controlling oxidative stress and inflammation. These effects were supported by coordinated biochemical, molecular, and histological findings, alongside consistent changes in dielectric parameters. Dielectric spectroscopy provided complementary ex vivo biophysical information reflecting tissue structural and compositional alterations rather than in vivo electrophysiological function. Collectively, the results support apigenin as a promising natural compound for mitigating obesity-associated liver dysfunction and highlight dielectric spectroscopy as a useful adjunct tool in preclinical NAFLD research.

## Data Availability

Data is available when requested from the corresponding author.
